# Chemical-state distributions in charged LiCoO_2_ cathode particles visualized by soft X-ray spectromicroscopy

**DOI:** 10.1038/s41598-023-30673-1

**Published:** 2023-03-21

**Authors:** Wenxiong Zhang, Eiji Hosono, Daisuke Asakura, Hayato Yuzawa, Takuji Ohigashi, Masaki Kobayashi, Hisao Kiuchi, Yoshihisa Harada

**Affiliations:** 1 Institute for Solid State Physics (ISSP), The University of Tokyo, 5-1-5 Kashiwanoha, Kashiwa, Chiba 277-8581 Japan; 2 Global Zero Emission Research Center, National Institute of Advanced Industrial Science and Technology (AIST), 16-1 Onogawa, Tsukuba, Ibaraki 305-8569 Japan; 3 Research Institute for Energy Conservation, National Institute of Advanced Industrial Science and Technology (AIST), 1-1-1 Higashi, Tsukuba, Ibaraki 305-8565 Japan; 4 AIST-UTokyo Advanced Operando-Measurement Technology Open Innovation Laboratory, 5-1-5 Kashiwanoha, Kashiwa, Chiba 277-8565 Japan; 5 UVSOR Synchrotron Facility, Institute for Molecular Science, Okazaki, 444-8585 Japan; 6 Department of Electrical Engineering and Information Systems, The University of Tokyo, 7-3-1 Hongo, Bunkyo-Ku, Tokyo, 113-8656 Japan; 7 Center for Spintronics Research Network, The University of Tokyo, 7-3-1 Hongo, Bunkyo-Ku, Tokyo, Japan

**Keywords:** Electrochemistry, Batteries, Electronic properties and materials, Microscopy

## Abstract

Lithium-ion deintercalation/intercalation during charge/discharge processes is one of the essential reactions that occur in the layered cathodes of lithium-ion batteries, and the performance of the cathode can be expressed as the sum of the reactions that occur in the local area of the individual cathode particles. In this study, the spatial distributions of the chemical states present in prototypical layered LiCoO_2_ cathode particles were determined at different charging conditions using scanning transmission X-ray microscopy (STXM) with a spatial resolution of approximately 100 nm. The Co *L*_3_- and O *K*-edge X-ray absorption spectroscopy (XAS) spectra, extracted from the same area of the corresponding STXM images, at the initial state as well as after charging to 4.5 V demonstrate the spatial distribution of the chemical state changes depending on individual particles. In addition to the Co *L*_3_-edge XAS spectra, the O *K*-edge XAS spectra of the initial and charged LiCoO_2_ particles are different, indicating that both the Co and O sites participate in charge compensation during the charging process possibly through the hybridization between the Co 3*d* and O 2*p* orbitals. Furthermore, the element maps of both the Co and O sites, derived from the STXM stack images, reveal the spatial distribution of the chemical states inside individual particles after charging to 4.5 V. The element mapping analysis suggests that inhomogeneous reactions occur on the active particles and confirm the existence of non-active particles. The results of this study demonstrate that an STXM-based spatially resolved electronic structural analysis method is useful for understanding the charging and discharging of battery materials.

## Introduction

Lithium-ion batteries (LIBs) are the most used energy storage devices for portable electronics, electric vehicles and airplanes, and stationary devices. However, LIBs still exhibit several limitations related to low energy densities, poor long-cycling stability, and safety issues. As a result, significantly improving the electrochemical performance of LIBs is essential to satisfy the increasing demand for electric vehicles and smart grid systems^1–4^. In particular, remarkable improvements in cathode materials for LIBs are imperative to mitigate the aforementioned shortcomings. The performances of most cathode materials are limited by Li-ion deintercalation (charge reaction)/intercalation (discharge reaction), which occurs on the cathode particle surface, and the Li-ion diffusion rate in each particle. Therefore, analyzing individual particles present in a cathode material is essential to understand their chemical-state distributions during the charge/discharge processes. This information is expected to provide valuable guidelines for the development of novel cathode materials and modification of the typical ones to achieve record high capacities and excellent stabilities. In this study, we focused on the observation of individual particles of LiCoO_2_^5,6^, which is a commercially available layered cathode material for LIBs^7–10^. Previous reports suggested that the redox reaction of Co^3+^/Co^4+^ occurs in LiCoO_2_ accompanied with Li-ion deintercalation/intercalation^11–14^, and some researchers have highlighted the importance of the Co 3*d*–O 2*p* orbital hybridization^15–18^. However, the distribution of the Co^3+^/Co^4+^ during the redox reaction, i.e. the chemical-state distribution on local parts of each particle, is still unknown. In addition, distinguishing between the inactive and redox particles based on the spatial distribution of the chemical states is crucial.

X-ray absorption spectroscopy (XAS) is frequently employed to analyze the electronic structure of the electrode materials used in LIBs. XAS is sensitive to the valence states, bond covalencies, and spin states of selected elements^19–25^. In particular, soft X-rays can be used to probe transition metal (TM) 3*d* states through the allowed 2*p*-to-3*d* dipole transition, which quantitatively elucidates the redox-reaction-induced changes in the valence states upon electrochemical cycling^26–31^. Furthermore, the O *K*-edge XAS spectra reveals the changes in the electronic states of both O and TM ions based on the hybridization between the TM 3*d* and O 2*p* orbitals^26,32–34^. Co *L*-edge and O *K*-edge XAS spectral analyses indicate that both the Co and O ions in the LiCoO_2_ cathode particles participate in the redox process^35^, although the changes in the microscopic chemical-state distributions of each element remain unexplored.

Xu et al. demonstrated the inhomogeneous charge distribution in individual LiCoO_2_ particles by analyzing the Co *K*-edge spectra obtained by in situ transmission X-ray spectromicroscopy using hard X-rays^36^. However, identifying the oxidation states of TM oxides is a considerable challenge because of the weak signal originating from the forbidden 1*s*-to-3*d* dipole transition in TMs^32,37–39^. Therefore, a more elaborate analytical method that features high spatial and energy resolutions as well as a high sensitivity to TM 3*d* orbitals and hybridized oxygen valence orbitals is required to determine the Li-ion deintercalation/intercalation-induced changes in the electronic structures of individual cathode particles.

To solve this problem, we used soft X-rays to perform scanning transmission X-ray spectromicroscopy (STXM) analysis of LiCoO_2_ cathode particles. Soft X-ray STXM combines XAS with microscopy and is a powerful tool to determine chemical-state distributions owing to its high energy and spatial resolutions and excellent sensitivity to TM 3*d* orbitals and valence orbitals of light elements^40–46^. The STXM observations revealed the occurrence of inhomogeneous reactions inside individual charged particles. Herein, we use the STXM results to discuss the mechanism underlying the observed contribution of the Co 3*d*–O 2*p* hybridization to the oxidation reactions occurring on each area of a particle.

## Results and discussion

**Figure 1:**
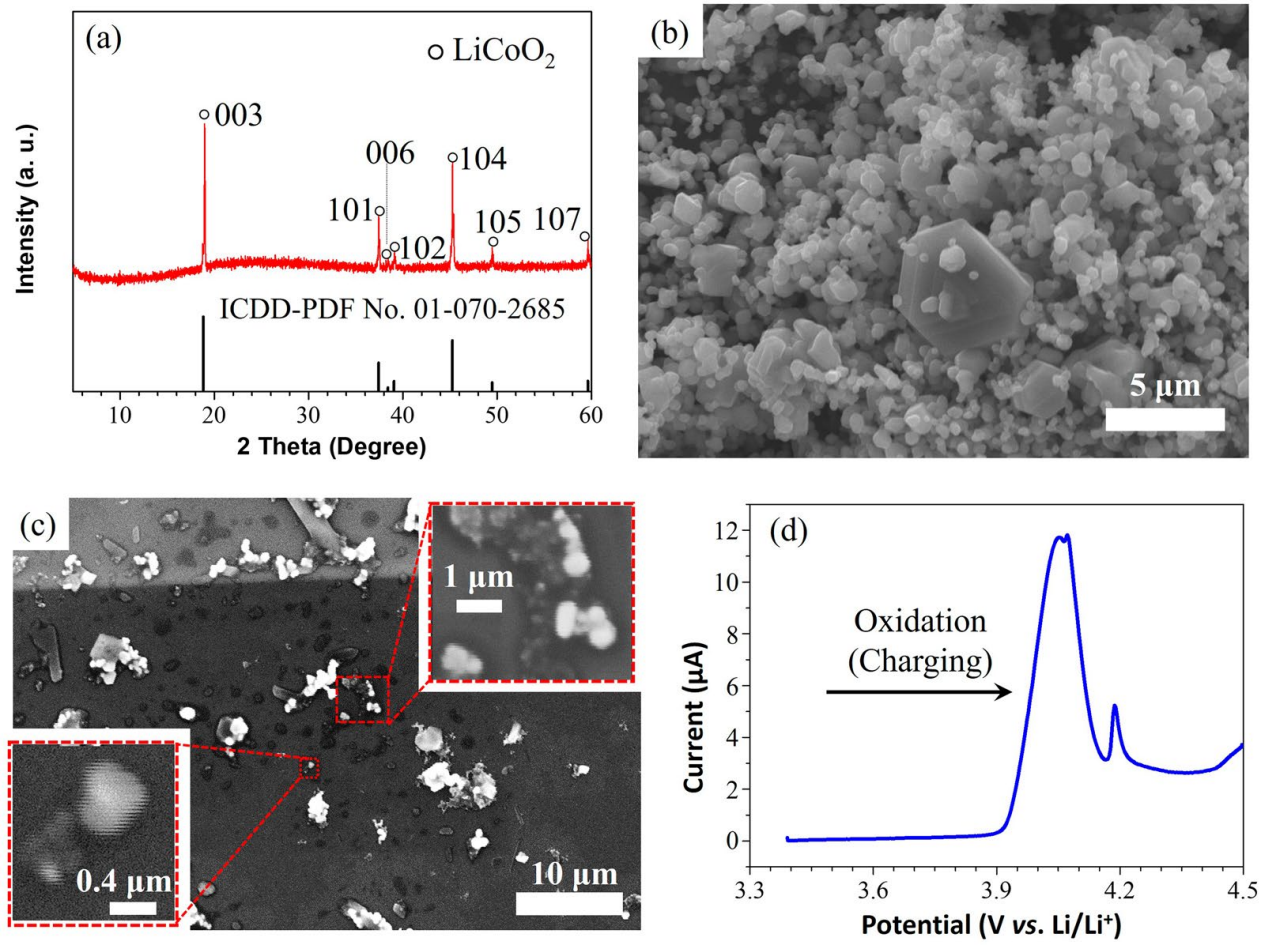
(**a**) XRD pattern and (**b**) SEM image of the LiCoO_2_ powder sample prepared by the molten salt method, and (**c**) SEM images of the LiCoO_2_ particles dropped on the working electrode for the STXM measurements. The insets show enlarged images of the particles investigated in this study. (**d**) Linear sweep voltammogram of LiCoO_2_ up to a cutoff voltage of 4.5 V.

Figure 1a shows the X-ray diffraction (XRD) pattern of the LiCoO_2_ particles, indicating the formation of the pure LiCoO_2_ phase belonging to the rhombohedral space group (*R*-3* m*). The separation of the (006)/(102) diffraction peaks reflects the presence of a layered structure of LiCoO_2_. The lattice constants a = 2.816 Å and c = 14.053 Å along the (100) and (001) directions, estimated from the pattern, are consistent with a = 2.816 Å and c = 14.054 Å obtained from the ICDD pattern (No. 01–070-2685)^47^. Figure 1b shows a scanning electron microscopy (SEM) image of the LiCoO_2_ particles with a size distribution ranging from several hundred nanometers to five micrometers. Figure 1c shows the SEM images of the LiCoO_2_ particles dispersed on the working electrode. All the particles shown in the inset images were evaluated by STXM. Figure 1d shows the linear sweep voltammogram of the LiCoO_2_ particles shown in Fig. 1c; the oxidation peak of LiCoO_2_ (4.05 V) appears during the charging to 4.5 V (vs. Li/Li^+^), suggesting the electrochemical Li-ion deintercalation of the LiCoO_2_ host^48^.

**Figure 2:**
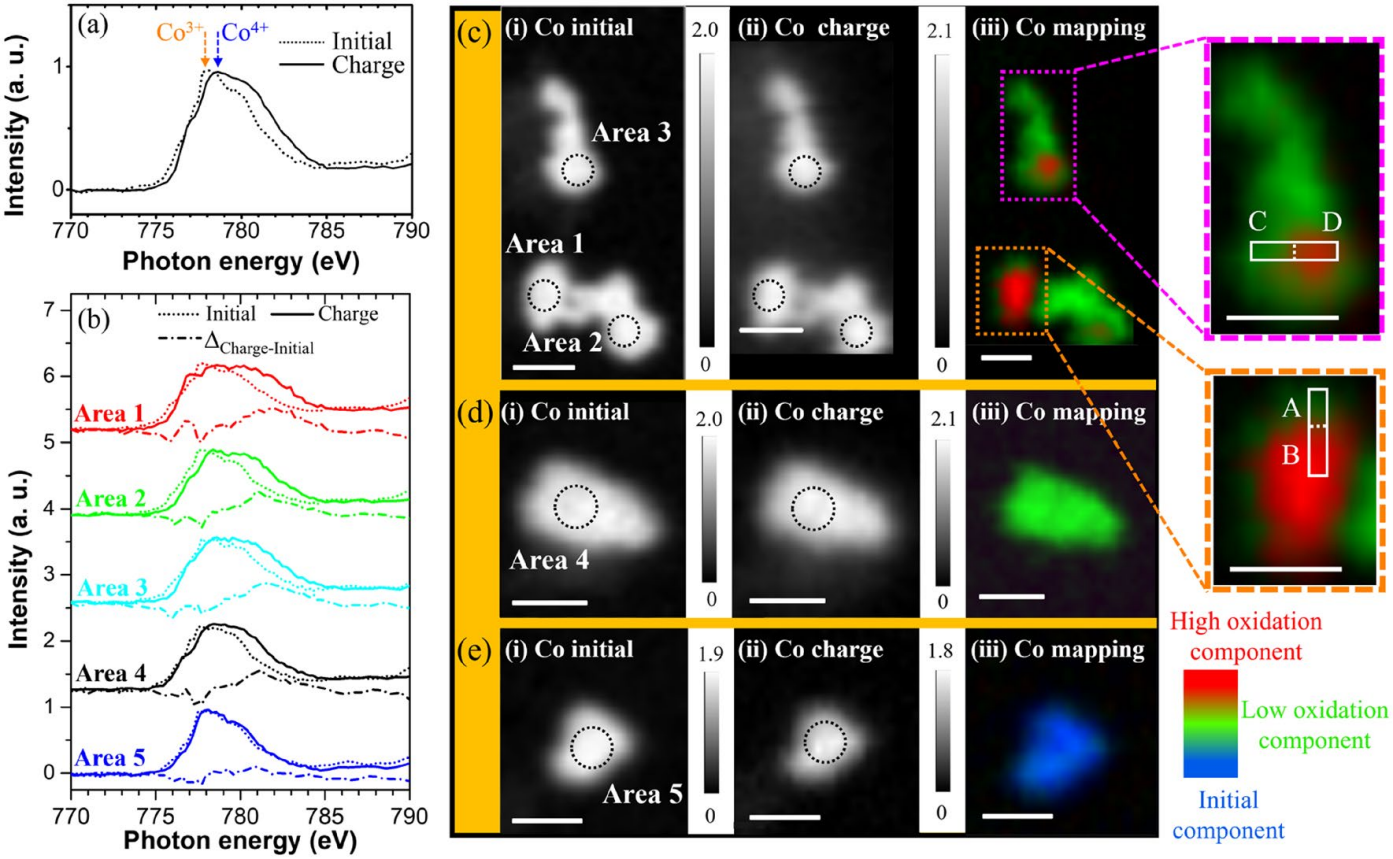
Co *L*_3_-edge XAS spectra of the initial (dotted line) and charged (solid line) states measured by STXM: (**a**) those averaged over all the particles and (**b**) those recorded at the circled areas (diameter: 300 nm) of the different LiCoO_2_ particles in (**c**)**,** (**d**) and (**e**). Also contained in (**b**) are the difference spectra (dash-dotted lines). (**c–e**) STXM OD images of the Co *L*_3_-edge of the LiCoO_2_ particles obtained at the (i) initial and (ii) charged states, and (iii) stack mappings (770–790 eV) shown by red, green, and blue colors, each indicating the charged states assigned according to the reference spectra (solid lines) obtained from Areas 1 and 2, and the initial state assigned using the reference spectrum (dotted line) obtained from Area 5 shown in (**b**). Enlarged rectangular regions (A, B, C, and D) separated by dashed lines are shown on the right. The red and green colors represent different oxidation states. All the OD images of Co are plotted at 778 eV, and the white scale bars represent 0.5 μm.

Figure 2a shows the Co *L*_3_-edge XAS spectra obtained from the STXM measurements, which are averaged over all the particles in the inset images shown in Fig. 1c, before and after the charging. The XAS profile of the initial state indicates the presence of the Co^3+^ (3*d*^6^) valence state in LiCoO_2_^35^. After charging to 4.5 V, the XAS spectral line shape is broadened toward higher energies. This change in the spectral line shape indicates the partial oxidation of the Co ions to Co^4+^ (3*d*^5^) because of the electrochemical Li-ion deintercalation of the LiCoO_2_ host^18^. Figure 2b shows the Co *L*_3_-edge XAS spectra recorded from the circled areas of the individual LiCoO_2_ particles in the STXM optical density (OD) images (Areas 1 to 5) shown in Figs. 2c–e for the initial and charged states. Note that the spectral line shape of the charged state recorded at Area 1 is different from those recorded at the other marked areas, whereas those of the initial states are nearly identical. In addition, the difference spectra (dash-dotted lines) of the initial and charged states recorded from marked areas are shown. Larger spectral intensity of them  at higher energies suggests that a larger number of oxidized Co species are produced because of the charging process, i.e. the ratio of the number of charged Co species to that of the initial Co species increases. Thus, a comparison between the difference spectra indicates that the amount of the oxidized Co^4+^ species induced by the Li-ion deintercalation in Area 1 is larger than that in the other areas. In contrast, the XAS profile recorded at Area 5 remains almost unchanged during the charging, suggesting that the redox reaction is not active on this area.

Next, we mapped the spatial distribution of the different oxidation states using the Co *L*_3_-edge XAS spectra. We selected two spectra (solid lines) obtained from Areas 1 and 2 with different spectral line shapes as the references of different electronic structures, and recorded one spectrum (dotted line) at Area 5 for the initial state. Subsequently, we fitted the full area by a linear combination of these three references. Figures 2c-iii, d-iii and e-iii show color maps, which indicate the contribution of the spectra obtained from Areas 1 (red), 2 (green), and 5 (blue) according to the portion of each color; this map is a stack mapping image. In these maps, the color contrast reflects the degree of oxidation, and the red, green, and blue colors represent a high oxidation state, a low oxidation state, and the initial state, respectively. As shown in Fig. 2c-iii, the predominant chemical states are different depending on the particles. Notably, the color contrasts on these particles are inhomogeneous (see the particles surrounded by squares), indicating that the particles react inhomogeneously during the charging. In contrast, the chemical state of the particle shown in Fig. 2e-iii is almost unchanged during the charging, suggesting that the particle is inactive for the redox reaction on the Co site.

**Figure 3:**
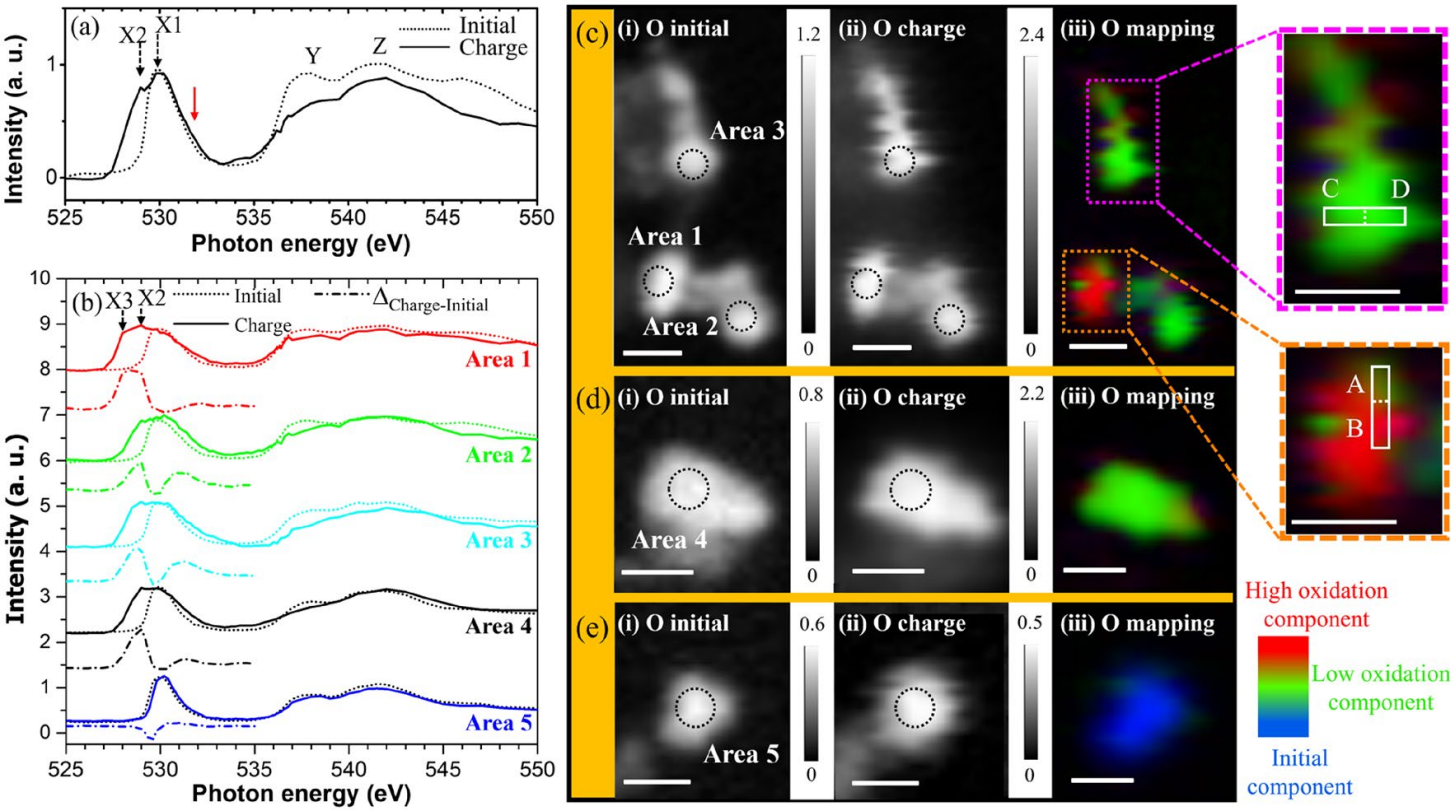
O *K*-edge XAS spectra of the initial (dotted line) and charged (solid line) states measured by STXM: (**a**) those averaged over all the particles and (**b**) those recorded at the circled areas (diameter: 300 nm) of different LiCoO_2_ particles in (**c**), (**d**) and (**e**). Also contained in (**b**) are the difference spectra (dash-dotted lines, 525–535 eV). (**c**–**e**) STXM OD images of O *K*-edge of the LiCoO_2_ particles obtained at the (i) initial and (ii) charged states, and (iii) stack mappings (525–550 eV) shown by red, green, and blue colors, each indicating the charged states assigned using the reference spectra (solid lines) of Areas 1 and 2, and the initial state assigned according to the reference spectrum (dotted line) recorded at Area 5 shown in (**b**). Enlarged rectangular regions (A, B, C, and D) separated by dashed lines are shown on the right. The red and green colors represent different oxidation states. All the OD images of O are plotted at 529 eV, and the white scale bars represent 0.5 μm.

Figure 3a shows the O *K*-edge XAS spectra similar to the Co *L*_3_-edge XAS (Fig. 2a). The X1 peak at 530 eV for the initial state corresponds to the transition of an O 1*s* electron to the O 2*p* orbitals hybridized with the Co 3*d* orbitals, whereas the Y and Z peaks correspond to the transition of an O 1*s* electron to the O 2*p* orbitals hybridized with the Co 4*s*/4*p* orbitals^35^. At the charged state, a new pre-edge peak appears at 529 eV marked as X2. Theoretical calculations of the O *K*-edge XAS spectrum confirm a significant contribution of TM 3*d* orbitals to the pre-edge peaks through the TM 3*d*–O 2*p* hybridization^49^. According to a previous report, the O *K* pre-edge variations are dominated by changes in the TM-*d* states^50^.

Similar to the Co *L*_3_-edge spectra, Fig. 3b shows the O *K*-edge XAS spectra of the initial and charged states, and their difference spectra recorded at the circled areas of the individual LiCoO_2_ particles visible in the STXM OD images in Figs. 3c–e. The pre-edge structures in the O *K*-edge XAS spectra change because of the charging, and these changes are different among the particles. Especially, the pre-edge peak is the most enhanced in the spectrum measured at Area 1 after the charging. The structure marked by X3, below X2, reflects the transition to the O 2*p* orbital hybridized with the t_2g_ orbital, where the charging reactions lead to the production of holes^18,35^. The broadened pre-edge structure suggests the formation of a higher oxidation state of LiCoO_2_ during the charging. The results indicate that among the marked Areas, Area 1 is the most active for charging because of the largest spectral line shape difference between the initial and charged states. Conversely, no charging-induced change is observed in the spectrum measured at Area 5, suggesting an inactive redox reaction. Figures 3c-iii, d-iii and e-iii show the stack mapping results of the O *K*-edge XAS spectra (solid lines) recorded at Areas 1 and 2 and that (dotted line) obtained from Area 5. Similar to the Co analysis, the particles in Fig. 3c-iii show different colors among themselves and even within each particle (see the areas surrounded by the rectangles), indicating that the charge distribution for the O component is also inhomogeneous. The chemical state of the particle shown in Fig. 3e-iii is almost independent of the charging process, suggesting that the particle is inactive even for the O-site redox reaction. The inactive redox process observed on one particle could be due to the poor electronic contact between the particle and the current collector. By contrast, the particles exhibiting charging-induced state changes due to their different activity will be discussed later.

Notably, the chemical-state distributions of the O components are mostly identical to those of the Co components. This provides experimental evidence that the O components in the LiCoO_2_ particles play a crucial role in the redox process via the hybridization with the Co 3*d* orbitals. The STXM observations reveal the presence of active and inactive particles. It is known that a LiCoO_2_ particle is composed of different facets, and the stability and diffusion rate of the Li-ions on each facet are different. The equilibrium shapes of the LiCoO_2_ and Li(Ni_1/3_Mn_1/3_Co_1/3_)O_2_ particles feature the (001), (104), and (012) surface facets^51,52^. It has been reported that the cycle performance of the (012) surface-dominated particles is less stable than that of the (001) surface-dominated particles^53^. Depending on the ratios of the different facets on the surface of each particle, the chemical state inhomogeneously changes inside the particle upon Li-ion diffusion. Thus, the chemical-state distributions inside the charged LiCoO_2_ particles can be ascribed to the different reactivities of the LiCoO_2_ particle surfaces.

**Figure 4:**
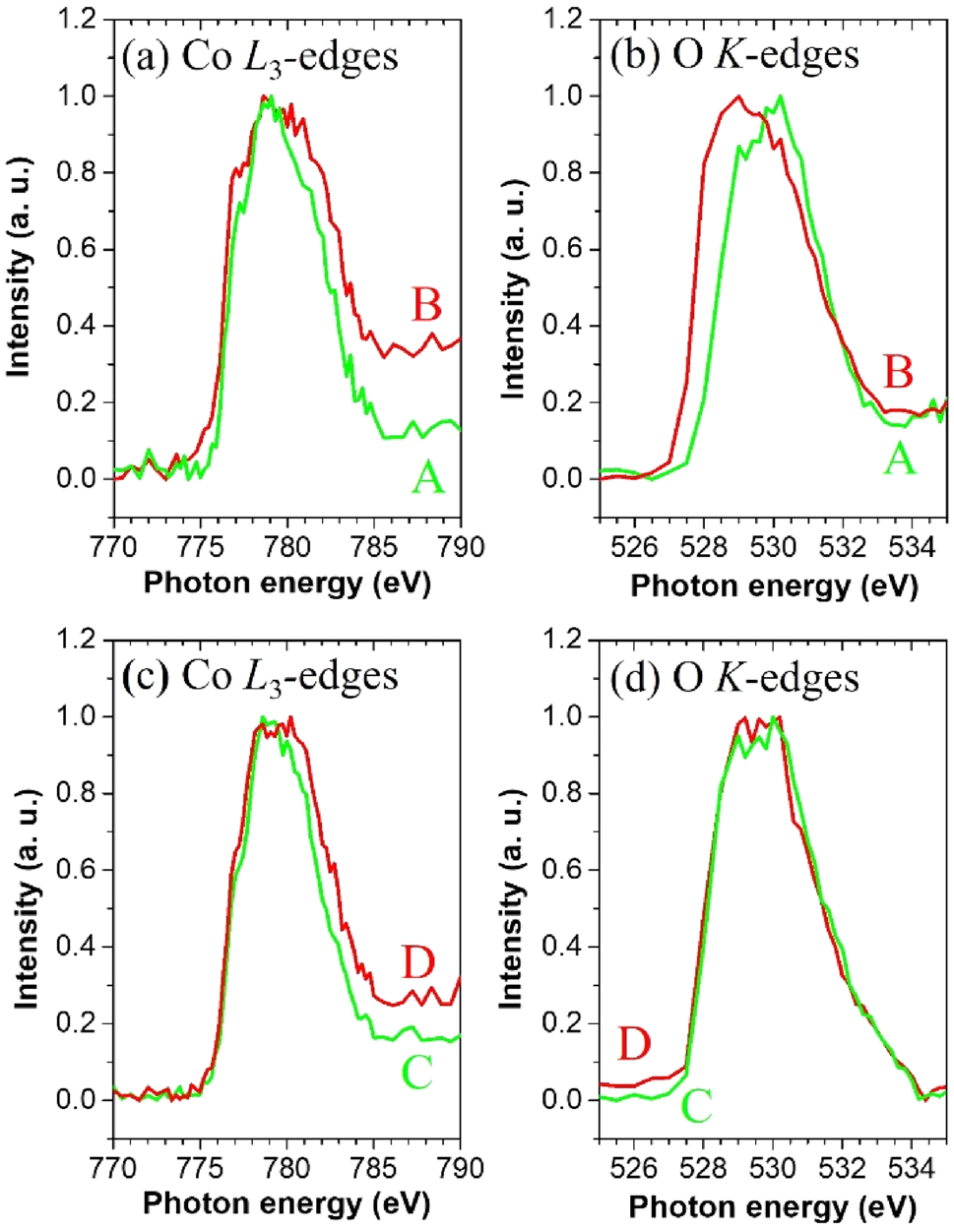
(**a**, **c**) Co *L*_3_-edge and (**b**, **d**) O *K*-edge spectra extracted from the local rectangular regions (A, B, C, and D) separated by dashed lines shown in Figs. 2c-iii and 3c-iii after charging to 4.5 V, respectively.

To explore the particle-dependent inhomogeneous chemical-state distributions in more detail, XAS spectra were derived from the non-uniform contrasts (regions A, B, C, and D) visible in the enlarged STXM stack maps shown in Figs. 2c-iii and 3c-iii. In Figs. 4a and b, the XAS spectra obtained from region B show increasing oxidation signals for both the Co and O components, whereas no increasing signal is evident in the spectrum obtained from region A. Thus, in this particle, the redox reaction should dominantly occur in the hybridized states between the Co 3*d* and O 2*p* orbitals. Another case is shown in regions C and D. In Figs. 4c and d, the XAS profiles show different oxidation properties of Co (inhomogeneous) and O (homogeneous), which possibly originate from the electronic structure involved in the charging process. It is known that the hybridization between the Co 3*d* and O 2*p* orbitals in LiCoO_2_ becomes stronger with Li-ion deintercalation during charging^54,55^. Considering that the diffusion rate of the Li ions depends on the facets of the particle, the differences between the chemical-state distributions of the Co and O components may be elucidated using the morphology of LiCoO_2_.

Notably, the STXM technique with a high spatial resolution aided in analyzing the electronic structural changes in Co and O at the same area of the LiCoO_2_ particle; such an analysis cannot be performed by conventional XAS, which provides only averaged electronic structural information^35^. Using the combined XAS and STXM-assisted mapping analyses, we successfully examined the different chemical states of individual LiCoO_2_ particles with charging up to 4.5 V. The corresponding results revealed the occurrence of inhomogeneous reactions among the particles as well as inside a particle. In this study, our investigations were limited to *ex-situ* samples, the electrochemical cycling-induced electronic structural changes in the LiCoO_2_ particles could not be evaluated. Nevertheless, the presence of an inhomogeneous Li-ion distribution and inactive chemical-state areas in a single LiCoO_2_ particle during the cycling process was verified in a recent study^36^. Furthermore, the cycling performance of LiCoO_2_ was found to depend on the particle size^56^. To address these issues, the morphology and size of the synthesized particles need to be controlled, and/or surface coating treatments should be applied. Additional *operando* measurements by STXM will be conducted in the near future using a newly designed *operando* battery cell, and a more detailed analysis of the cycling performance of LiCoO_2_ and other layered cathode particles will be performed.

## Conclusions

We studied the electronic structure of LiCoO_2_ particles using STXM before and after charging to 4.5 V. Using the STXM technique, we successfully visualized the local area of the individual LiCoO_2_ particles with a high spatial resolution of 100 nm and performed both spectroscopic and microscopic analyses. The Co *L*_3_-edge and the O *K*-edge XAS spectra showed the electronic structure of the individual LiCoO_2_ particles in their initial states and after charging. The STXM mapping analysis of both Co and O revealed the size- or morphology-dependent chemical-state changes in the particles. Furthermore, inhomogeneous reactions inside individual LiCoO_2_ particles were also revealed; in one region both Co and O were involved in the charge compensation owing to the strong hybridization of the Co 3*d* and O 2*p* orbitals, while in another region, mainly Co was found to be involved in the charge compensation. These advantages of the STXM technique will provide a novel protocol to improve the electrochemical performance of LiCoO_2_ and other layered cathodes via surface coating or morphology and size control of the individual particles of electrode materials. In addition, by combining STXM with the X-ray photoemission electron microscopy technique, which can be used to analyze the surface electronic structure of electrode materials, the redox reactions that occur both on the surface and inside the bulk of the electrode materials can be examined. This combined method is expected to aid in analyzing more integrated electronic states.

## Method

The LiCoO_2_ powder sample was synthesized by the molten salt method. NaCl, LiOH·H_2_O, and Co(NO_3_)_2_·6H_2_O were mixed and heated at 900 °C for 1 h in air. Finally, the samples were washed with distilled water and dried in vacuum.

The electrochemical measurements were performed using a three-electrode beaker cell (Fig. S1). A working electrode of the beaker cell was prepared from a current collector (Au-deposited Si_3_N_4_ membrane) and a LiCoO_2_ slurry. The current collector (7.5 × 7.5 mm^2^ area) consisted of Au (10 nm), Ti (3 nm), and Si_3_N_4_ (100 nm) multilayer films deposited on a Si frame (200 μm thick) (NORCADA, NX7100C). The central region of this membrane chip (1 × 1 mm^2^) acted as the window of the multilayer films and permitted the transmission of soft X-rays during the STXM measurements. The slurry was prepared by ultrasonically mixing LiCoO_2_, super P Li (electro-conductive additive), and N-methyl-2-pyrrolidone. The prepared slurry was then dropped on the membrane using a pipette and dried at 100 °C for 5 min. Then, the cell was assembled inside an Ar-filled glovebox. Li metal was pressed onto the Cu current collectors to serve as both counter and reference electrodes of the beaker cell. A 1 M LiClO_4_ ethylenecarbonate/diethyl-carbonate (EC/DEC) solution (1:1 v/v %) was used as the electrolyte. The cutoff voltage was set to 4.5 V for the linear sweep voltammetry (Biologic, VMP3) measurements with a sweep speed of 0.5 mV/s. After charging, the cells were rapidly disassembled, and the working electrode was washed with the EC/DEC solution inside the Ar-filled glovebox and subsequently dried in vacuum for 5 h. Finally, the Si-chip electrode was transferred into the STXM chamber using a transfer vessel without air exposure. Note that the selected LiCoO_2_ particles on the Si-chip electrode were evaluated by STXM before applying the voltage and again after the charging process.

The structure of the powder sample was investigated using a powder X-ray diffractometer and Cu Kα radiation (Bruker, D2 PHASER). The particle size and morphology of the sample were observed using a scanning electron microscope (JEOL, JCM-6000PLUS).

The STXM measurements were performed using the STXM apparatus installed at the BL4U beamline of the UVSOR-III Synchrotron Facility, Institute for Molecular Science, Okazaki, Japan^40^. The sample was raster-scanned using a focused beam to form a 2D image of the XAS spectra in transmission mode. The image in each sequence of the excitation energy, referred to as an image stack, was aligned by a spatial cross-correlation analysis performed using the aXis2000 software^57^. The XAS spectra was then extracted from any pixel or group of pixels of the stack. Typical sequence images were obtained with a view using a 100 × 100 nm^2^ pixel size. Two areas were selected in the OD image, where the spectral line shapes of the Co *L*_3_-edge and O *K*-edge XAS profile changed differently in the initial and charged states, and the mapping analysis of the entire image was performed using the stack fit tools assembled in aXis2000, and these chemical states were used as a reference. Using the stack fitting results, we derived the number of valence states for each element^58–61^.

## Supplementary Information

Supplementary Figure S1.

## Data Availability

All data generated or analyzed during this study are included in this article, and the datasets used or analyzed during the current study are available from the corresponding author with reasonable request.
