# Supplementary material for: Chemical-state distributions in charged LiCoO2 cathode particles visualized by soft X-ray spectromicroscopy
Source: Sci Rep. 2023 Mar 21;13:4639. doi: 10.1038/s41598-023-30673-1 (PMC10030574; doi:10.1038/s41598-023-30673-1)
Supplement: Supplementary file 1 — Supplementary Figure S1. [file 41598_2023_30673_MOESM1_ESM.docx]

**Supporting information**

**Chemical-state distributions in charged LiCoO_2_ cathode particles visualized by soft X-ray spectromicroscopy**

Wenxiong Zhang^a^, Eiji Hosono^b,c,d*^, Daisuke Asakura^b,c,d^, Hayato Yuzawa^e^, Takuji Ohigashi^e^, Masaki Kobayashi^f,g^, Hisao Kiuchi^a^, Yoshihisa Harada^a,d,h*^

^a^ Institute for Solid State Physics (ISSP), The University of Tokyo, 5-1-5 Kashiwanoha, Kashiwa, Chiba 277-8581, Japan

^b^ Global Zero Emission Research Center, National Institute of Advanced Industrial Science and Technology (AIST), 16-1 Onogawa, Tsukuba, Ibaraki 305-8569, Japan

^c^ Research Institute for Energy Conservation, National Institute of Advanced Industrial Science and Technology (AIST), 1-1-1 Higashi, Tsukuba, Ibaraki 305-8565, Japan

^d^ AIST-UTokyo Advanced Operando-Measurement Technology Open Innovation Laboratory, 5-1-5 Kashiwanoha, Kashiwa, Chiba 277-8565, Japan

^e^ UVSOR Synchrotron Facility, Institute for Molecular Science, Okazaki 444-8585, Japan

^f^ Department of Electrical Engineering and Information Systems, The University of Tokyo, 7-3-1 Hongo, Bunkyo-ku, Tokyo 113-8656, Japan

^g^ Center for Spintronic Research Network, The University of Tokyo, 7-3-1 Hongo, Bunkyo-ku, Tokyo

^h^ Synchrotron Radiation Research Organization, The University of Tokyo, 7-3-1 Hongo, Bunkyo-ku, Tokyo, 113-8656, Japan

*Corresponding authors

Yoshihisa Harada:

Tel.: +81791-58-1973 (6620) E-mail address: harada@issp.u-tokyo.ac.jp

Eiji Hosono:

Tel.: +8129-861-5169 E-mail address: e-hosono@aist.go.jp


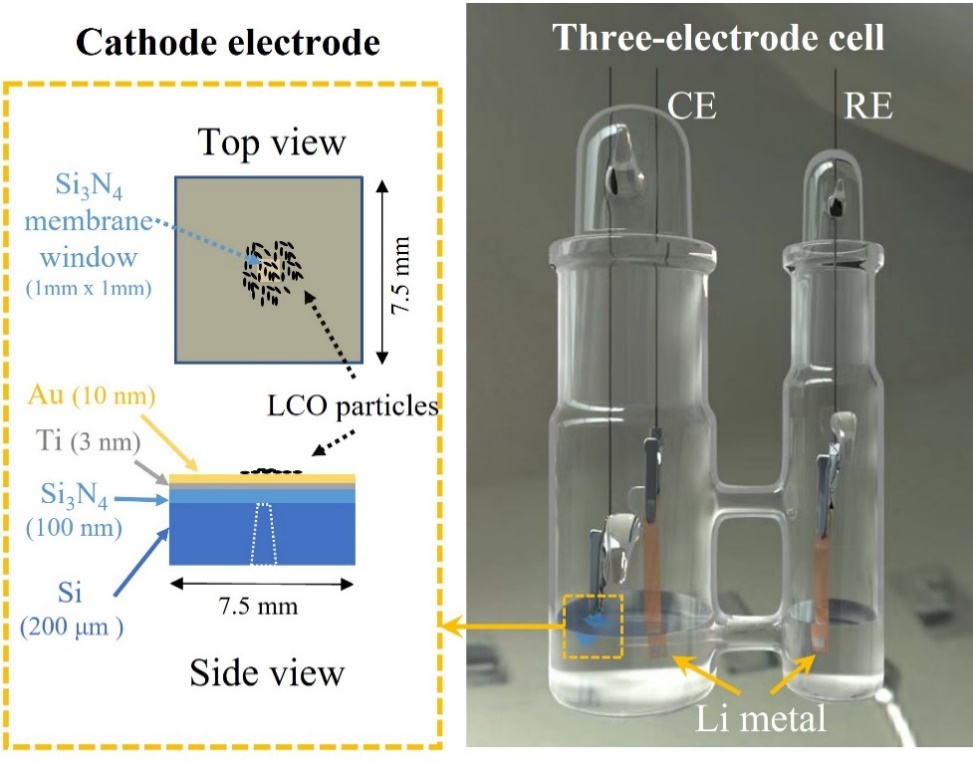


**Figure S1.** Schematic images for LiCoO_2_ cathode electrode and setup of electrochemical measurement by three-electrode beaker cell with Li metal as both counter electrode (CE) and reference electrode (RE) and 1 M LiClO_4_/EC/DEC solution as electrolyte.
